# Common themes in nutrient acquisition by plant symbiotic microbes, described by the Gene Ontology

**DOI:** 10.1186/1471-2180-9-S1-S6

**Published:** 2009-02-19

**Authors:** Marcus C Chibucos, Brett M Tyler

**Affiliations:** 1 Virginia Bioinformatics Institute, Virginia Polytechnic Institute and State University, Blacksburg, VA 24061, USA; 2 Current address: Institute for Genome Sciences, University of Maryland School of Medicine, Baltimore, MD 21201, USA

## Abstract

A critical function for symbionts is the acquisition of nutrients from their host. Relationships between hosts and symbionts range from biotrophic mutualism to necrotrophic parasitism, with a corresponding range of structures to facilitate nutrient flow between host and symbiont. Here, we review common themes among the nutrient acquisition strategies of a range of plant symbiotic microorganisms, including mutualistic symbionts, biotrophic pathogens that feed from living tissue, necrotrophic pathogens that kill host tissue, and hemibiotrophic pathogens that switch from biotrophy to necrotrophy. We show how Gene Ontology (GO) terms developed by the Plant-Associated Microbe Gene Ontology (PAMGO) Consortium can be used for describing commonalities in nutrient acquisition among diverse plant symbionts. Where appropriate, parallels found among animal symbionts are also highlighted.

## Symbiosis, a range of intimate relationships

Plants, animals, and diverse microbes engage in a wide range of interactions that can be characterized as symbiotic, that is, the living together of unlike organisms [1-5]. The Plant-Associated Microbe Gene Ontology (PAMGO) Consortium [6] has been developing an extensive set of Gene Ontology (GO) [7] terms that describe processes and structures underlying symbiotic interactions between organisms, ranging from mutualists through parasites [8]. This mini-review focuses on the nutrient acquisition strategies of a range of symbiotic organisms. Here "nutrient" is defined as any chemical substance required for metabolism or development. GO terms that describe gene products related to nutrient exchange during symbiosis are discussed along with examples of symbioses involving bacteria, protozoans, fungi, animals, oomycetes, algae, and plants.

## The Gene Ontology

The GO is a controlled vocabulary consisting of GO terms that describe gene product attributes in any organism [9]. GO terms are arranged as directed acyclic graphs (DAGs) within three ontologies, "GO: 0005575 cellular component", "GO: 0008150 biological process", and "GO: 0003674 molecular function". DAGs differ from hierarchies in that each term (child) may be related to more than one less specific term (parent). Three specific relationships among parent and child terms within a DAG are currently recognized by the GO: "is_a", "part_of", and "regulates". For example, "GO: 0052010 catabolism by symbiont of host cell wall cellulose" is a type of "GO: 0052009 disassembly by symbiont of host cell wall", and thus these terms would be connected by the "is_a" relationship (for more information on term-term relationships and ontology structure, see [9]).

## The concept of symbiosis in the Gene Ontology

**Figure 1:**
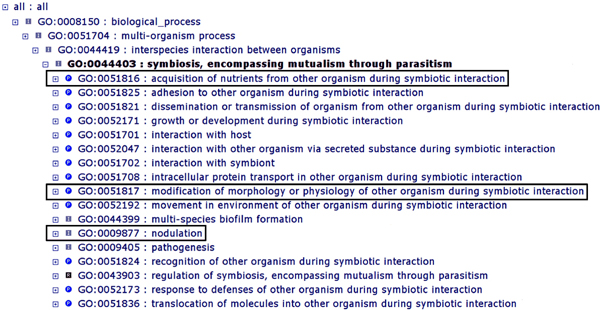
**Parent and child terms of "GO: 0044403 symbiosis, encompassing mutualism through parasitism" displayed in the AmiGO browser **[10]. "GO: 0044403 symbiosis, encompassing mutualism through parasitism" has several child terms that describe processes involved in nutrient exchange: "GO: 00051816 acquisition of nutrients from other organism during symbiotic interaction"; "GO: 0051817 modification of morphology or physiology of other organism during symbiotic interaction"; and "GO: 0009877 nodulation". These terms (highlighted by dark ovals), and selected child terms, can be seen in greater context in Figure 2. (Note that the numbers of gene products annotated to a given term, as typically displayed by AmiGO, have been removed for simplicity.)

**Figure 2:**
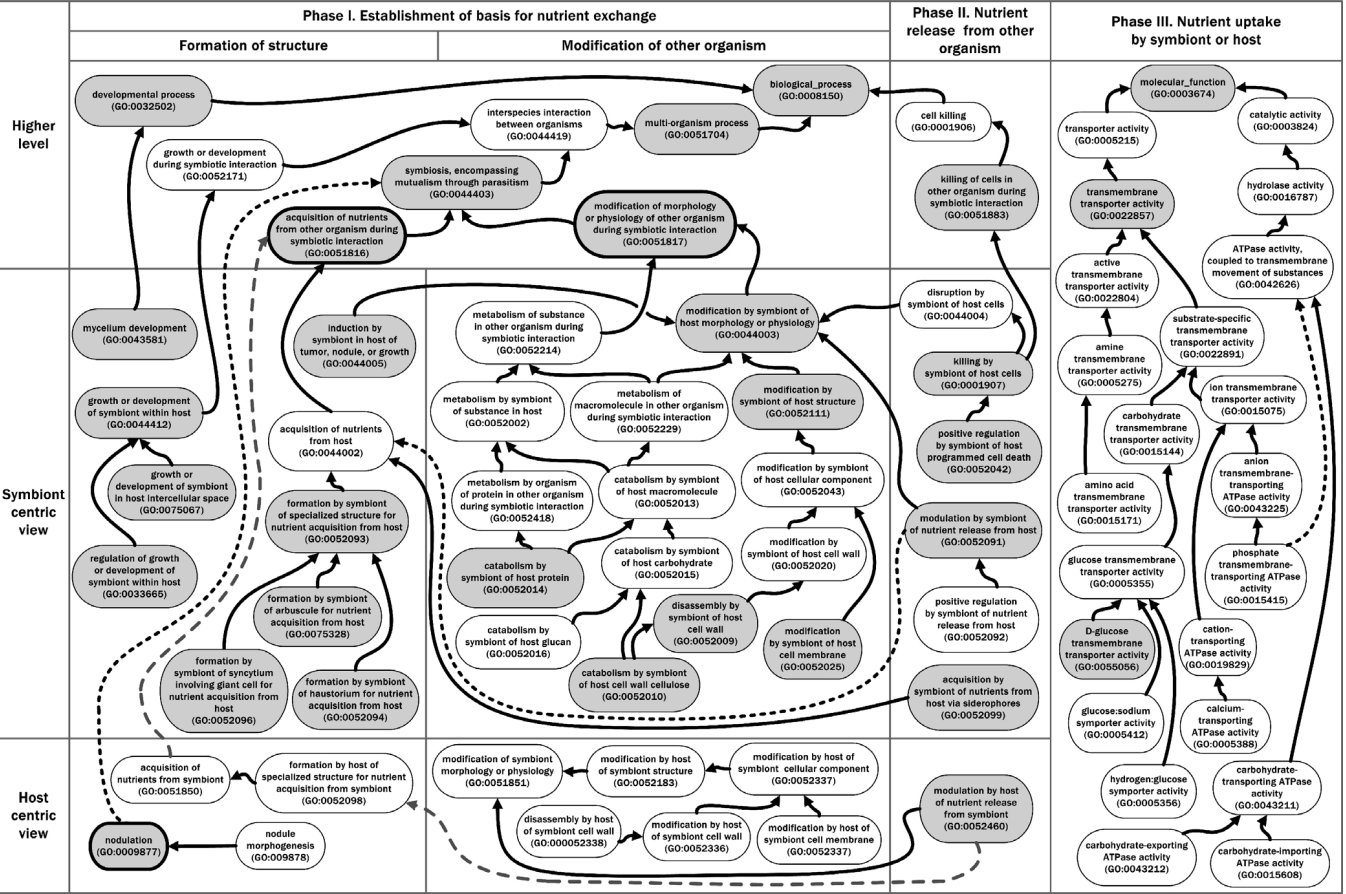
**Gene Ontology terms relevant to three phases of symbiotic nutrient exchange**. Processes associated with phases I and II of nutrient exchange are described by GO terms from the "GO: 0008150 biological_process" ontology. Terms at the top of the diagram describe higher level processes, terms in the middle represent symbiont processes, and terms at the bottom characterize host processes. Functions associated with phase III are described with GO terms from the "GO: 0003674 molecular_function" ontology that describe nutrient uptake irrespective of symbiotic partner. In the GO, term relationships take the form of a directed acyclic graph (DAG), similar to a hierarchy, except that a given term can have multiple parent terms or multiple child terms. Here, for simplicity, only selected terms are shown, and only a subset of the parent-child relationships are depicted; arrows symbolize GO "is_a" and "part_of" relationships (for more information on term relationships and other aspects ontology structure, i.e. "is_a", "part_of", and "regulates," see [9]). Some dashed arrows are used to enhance readability. GO terms highlighted by dark ovals represent GO terms also shown in Figure 1, and terms filled with grey can be found in the text.

In the GO, the concept of symbiosis is represented by the term "GO: 0044403 symbiosis, encompassing mutualism through parasitism", which is defined as: "An interaction between two organisms living together in more or less intimate association. The term host is usually used for the larger (macro) of the two members of a symbiosis. The smaller (micro) member is called the symbiont organism" [10]. The various forms of symbiosis include parasitism, in which the association is disadvantageous or destructive to the host organism; mutualism, in which the association is advantageous to both; and commensalism, in which the symbiont benefits while the host is not affected [8]. However, mutualism, parasitism, and commensalism are not discrete categories of interactions but rather a continuum. In fact, the nature of a symbiotic interaction may vary due to developmental changes in the host or symbiont, changes in the biotic or abiotic environment, or variation in host genotype [11]. Correspondingly, the exchange of nutrients between symbiotic partners may be context dependent and may be bidirectional or heavily unidirectional. The PAMGO Consortium strongly discourages the common but incorrect usage of the term "symbiosis" as a synonym for "mutualism" [8]. Figure 1 illustrates parent and child terms of "GO: 0044403 symbiosis, encompassing mutualism through parasitism", as viewed with the AmiGO browser [10]. Examples of child terms describing biological processes related directly or peripherally to nutritional exchange between symbionts and hosts include: "GO: 00051816 acquisition of nutrients from other organism during symbiotic interaction"; "GO: 0051817 modification of morphology or physiology of other organism during symbiotic interaction"; and "GO: 0009877 nodulation". These and other terms are described in greater detail in Figure 2 and Additional file 1.

## Symbiotic nutrient exchange

Strategies for nutrient exchange between host and symbiont organisms, which may involve formation of structures or modification of cell components of one or both organisms, are astonishingly diverse. For example, necrotrophic plant pathogens make nutrients available by producing enzymes that degrade host cell components including cell wall polysaccharides, e.g. "GO: 0052010 catabolism by symbiont of host cell wall cellulose", and cell membrane proteins, e.g. "GO: 0052025 modification by symbiont of host cell membrane" or "GO: 0052014 catabolism by symbiont of host protein" [12,13] (Figure 2). On the other hand, many biotrophic pathogens colonize host cells via haustoria, differentiated intracellular hyphal structures that facilitate nutrient uptake and suppression of host defenses [14], e.g. "GO: 0052094 formation by symbiont of haustorium for nutrient acquisition from host" (Figure 2 and explained below). Other interesting examples include: parasitic plants and algae [15]; mutualisms of lichenaceous fungi with cyanobacteria and/or green algae [16]; mutualisms of algae within the cytoplasm of protozoans [17]; and symbioses between coral polyps and dinoflagellate algae that are mutualistic or antagonistic depending on the ocean temperature [18]. Annotating gene products involved in symbiotic nutrient exchange with GO terms facilitates comparison among host and symbiont species from diverse kingdoms of life.

### Gene Ontology terms relevant to nutrient exchange, in a temporal framework

In Figure 2 we have represented the establishment of symbiotic nutrient exchange as occurring in three overlapping phases. Phase I involves establishing the physical basis for nutrient exchange through formation of structures or modification of the morphology or physiology of the other organism, or both. In phase II the release of nutrients from the symbiotic partners is achieved, for example through cell killing or modulation of nutrient release. Phase III comprises uptake of nutrients released in phase II, for example via transporters. Figure 2 summarizes GO terms relevant to symbiotic nutrient exchange within this temporal framework.

Terms from the Biological Process ontology related to symbiosis and cell killing are relevant principally to phases I and II, while many terms relevant to phase III are found in the Molecular Function ontology (Figure 2). The terms shown under phases I and II come from the "GO: 0051704 multi-organism process" branch of the Biological Process ontology that was created by PAMGO specifically to characterize symbiotic and other multi-organism interactions [8].

Phase I contains two important high-level GO terms, "GO: 0051816 acquisition of nutrients from other organism during symbiotic interaction" and "GO: 0051817 modification of morphology or physiology of other organism during symbiotic interaction". More specific child terms describe symbiont- or host-centric processes of morphological or physiological modification or structure formation; some of these terms are defined in Additional file 1.

Key terms within phase II that describe nutrient release from the other organism include "GO: 0051883 killing of cells in other organism during symbiotic interaction" [19], "GO: 0052091 modulation by symbiont of nutrient release from host", and "GO: 0052460 modulation by host of nutrient release from symbiont". All of those GO terms describe the process of making nutrients available for uptake by a symbiotic partner. In addition, terms such as "GO: 0052099 acquisition by symbiont of nutrients from host via siderophores" describe uptake of a (metal ion) nutrient that could occur through active interaction with the host, as described above, or through a passive mechanism such as acquisition from a plant root exudate by a microbe located in the rhizosphere [20].

Phase III of Figure 2 depicts representative terms from the Molecular Function ontology that describe transmembrane transporter-mediated uptake of nutrients. These terms describe attributes of gene products irrespective of symbiotic context. For example, "GO: 0055056 D-glucose transmembrane transporter activity" describes a gene product that transports glucose, whether that transport is part of an endogenous intra-organismal process or uptake following symbiotic killing of cells, e.g. "GO: 0051883 killing of cells in other organism during symbiotic interaction", and consequent release of glucose.

## Survey of symbiotic nutritional strategies

The following sections highlight mechanisms employed by diverse symbionts and hosts, both animal and plant, in order to facilitate nutrient exchange.

### Oomycetes and fungi: hyphae and haustoria

Oomycetes and fungi comprise two evolutionarily distinct groups, but share many commonalities with respect to morphology and ecological niche. Filamentous species from both groups include necrotrophic, biotrophic or hemibiotrophic pathogens of plants and animals that share common colonization strategies [21], including the early stages of infection from adhesion through penetration [22]. Hyphae are threadlike structures comprising the body of a filamentous organism through which nutrient uptake occurs. "GO: 0043581 mycelium development", a child of "GO: 0032502 developmental process" in the Biological Process ontology, describes the formation of a mass of hyphae (Additional file 1 and Figure 2). Many types of hyphae exist, including sub-cuticular (e.g. the fungus *Venturia inaequalis*), intercellular (e.g. the fungi *Cladosporium fulvum *and *Magnaporthe grisea *and the oomycete *Phytophthora sojae*), and intracellular (e.g. the fungus *Claviceps purpurea*, arbuscular mycorrhizal fungi, and the oomycete *Phytophthora infestans*) (reviewed in [22,23]). Some hemibiotrophs rely on intracellular hyphae which can spread from cell to cell [23]. Many obligate biotrophs, as well as some hemibiotrophs, generate modified hyphal infection structures known as haustoria [21-23] (e.g. the fungi *Uromyces appendiculatus*, *Erysiphe pisi*, and *Blumeria graminis*, and the oomycetes *Albugo candida *and *Phytophthora infestans*) that allow them to live in intimate contact with the host.

The haustorium is represented in the GO by the term "GO: 0052094 formation by symbiont of haustorium for nutrient acquisition from host" (Additional file 1 and Figure 2), a child of "GO: 0052093 formation of specialized structure for nutrient acquisition from host". This GO term is defined as "the assembly by an organism of a haustorium, a projection from a cell or tissue that penetrates the host's tissues for the purpose of obtaining nutrients from its host organism" [10]. In order to achieve this, the haustorium itself biosynthesizes materials [24], modulates host metabolism such as carbon sinks [25], and contributes to the suppression of host defenses [26-28]. Additional GO terms related to haustoria include: "GO: 0075192 haustorium mother cell formation on or near host"; "GO: 0075196 adhesion of symbiont haustorium mother cell to host"; and "GO: 0075197 formation of symbiont haustorium neck for entry into host".

Since haustoria are essential to many plant pathogens, plants have evolved active mechanisms to inhibit haustorium formation or to destroy haustorial cells via programmed cell death (reviewed in [29,30]). As a result, haustorium formation is accompanied by release of pathogen effector molecules that suppress plant defenses including programmed cell death (reviewed in [27,31] and in this supplement [32]).

One organism in which haustorium development and function have been well studied is the bean rust fungus *Uromyces fabae *[23,33]. During development of the haustorial body (reviewed in [22]), the host plasma membrane remains unbroken by the biotroph and undergoes extensive differentiation [34]. A complex mixture of metabolites, along with a modified symbiont cell wall, exists within the extrahaustorial matrix, the zone between the plant and fungal plasma cell membranes [35] where nutrient exchange occurs. Haustorial membranes exhibit increased H^+^-ATPase activity [36], which generates proton gradients that drive active transport of nutrients, including amino acids [37] and carbohydrates (reviewed in [33]).

Oomycetes such as *Phytophthora sojae *and *P. infestans *generate haustoria from intercellular hyphae [38]. As in biotrophs, the haustoria exhibit extensive modifications. For example, in the *P. sojae-*soybean interaction, the host membrane (the extrahaustorial membrane) exhibits different patterns of antibody labelling of arabinogalactan proteins than in nearby uninfected cells [39].

### Arbuscules of mutualistic arbuscular mycorrhizal fungi

In mutualistic symbioses such as the plant root-arbuscular mycorrhizal (AM) fungus association, nutrient exchange is bidirectional. In essence, the plant exchanges hexose sugars for inorganic phosphate from the fungal symbiont [40]. AM associations are very ancient and may have allowed plants to colonize land [41]. A variety of structures exist to facilitate nutrient exchange within the AM symbiosis, including arbuscules and hyphal coils that are formed within the cortical cells of the plant [42]. An arbuscule is a "fine, tree-like hyphal structure projected into the host cell for the purpose of obtaining nutrients from its host organism" [10]. Following establishment of the symbiosis, many genes associated with nutrient exchange are expressed by both host and symbiont [43]. For example, expression of fungal high affinity P_i _transporters in *Glomus *species depends on internal P_i _titer [44], and uptake of P_i _by the fungus and exchange with the host are regulated by plant carbon availability [45].

In the GO, terms addressing formation of arbuscules are children of "GO: 0075328 formation by symbiont of arbuscule for nutrient acquisition from host" (Additional file 1 and Figure 2) [10]. This term is a child of "GO: 0052093 formation of specialized structure for nutrient acquisition from host" and a sibling of terms such as "GO: 0052096 formation by symbiont of syncytium involving giant cell for nutrient acquisition from host" (see next paragraph) and "GO: 0052094 formation by symbiont of haustorium for nutrient acquisition from host", which underscores the potential for using this family of terms to facilitate cross kingdom functional comparisons of gene products involved in nutrient exchange. Further development of GO terms that describe such processes or structures is necessary. For example, there are a variety of categories of mycorrhizas, including AM, ectomycorrhizas, orchid mycorrhizas, and ericoid mycorrhizas [46]. New GO terms might address the formation of an ectomycorrhizal Hartig net, which allows for translocation of phosphorus in exchange for host carbohydrate [47]. In addition, there are commonalities in the signaling pathways of AM fungi and rhizobial bacteria in their mutualistic associations with legumes [48] that could be described by GO terms.

### Syncytia and giant cells in plant-nematode symbioses

Sedentary endoparasitic nematodes are biotrophic animal pathogens of diverse plant species, and include cyst nematodes and root-knot nematodes [49]. Cyst nematodes, including the economically important genera *Globodera *and *Heterodera*, produce highly specialized feeding structures known as syncytia that form via fusion of host cells. Root-knot nematodes including *Meloidogyne *species produce multinucleate giant cells by uncoupling host nuclear division from cell division. Syncytia and giant cells significantly differ from one another with respect to cellular structure, but both act as a nutrient sink, are multinucleated, hypertrophied cells with many vacuoles, and are highly metabolically active [50-52]. "GO: 0052096 formation by symbiont of syncytium involving giant cell for nutrient acquisition from host" (Additional file 1 and Figure 2) is a child term of "GO: 0052093 formation of specialized structure for nutrient acquisition from host". Additional GO terms exist that describe syncytium formation, including "GO: 0060140 syncytium formation by plasma membrane fusion of virally targeted cells", "GO: 0000768 syncytium formation by plasma membrane fusion", and several others [10].

### Bacterial nodules, galls, and endosymbionts

A huge diversity of bacterial symbionts colonize plants, animals, and even fungi [53]. Some of these are largely pathogenic, but many provide the host with essential services, including, for example, cellulose degradation, nitrogen metabolism, and fat metabolism in ruminant animals [54]. The GO currently has many terms that describe aspects of the mutualism between legumes and nitrogen fixing bacteria, including "GO: 0009877 nodulation" (Additional file 1, Figure 1, and Figure 2), defined as "the formation of nitrogen-fixing root nodules on plant roots" [10]. Other terms from the Cellular Component ontology describe the physical components of this mutualism, including "GO: 0043663 host bacteroid-containing symbiosome", defined as "a symbiosome containing any of various structurally modified bacteria, such as those occurring on the root nodules of leguminous plants, of a host cell" [10] (Additional file 1).

In contrast to mutualistic root nodulation, "GO: 0044005 induction by symbiont in host of tumor, nodule, or growth" is defined as "the process by which an organism causes the formation of an abnormal mass of cells in its host organism..." [10] (Figure 2). As a child term of "GO: 0044003 modification by symbiont of host morphology or physiology", this term could be used to describe the tumor-inducing activity of *Agrobacterium tumefaciens*, which results in plant galls [55].

There are many examples of bacterial endophytes, whose nutritional needs are met while supplying hosts with necessary nutrients or other benefits such as bioluminescence. The free-living, nitrogen-fixing bacterium *Acetobacter diazotrophicus*, which colonizes sugar cane, benefits from the low O_2 _levels and high sucrose levels necessary for nitrogenase activity [56]. In the symbiosis of the squid *Euprymna scolopes *and *Vibrio fischeri *bacteria, the bioluminescence of the bacteria, housed in a bilobed light organ, acts as an anti-predatory mechanism for the squid [57]. Symbiont-induced host tissue development leads to the formation of the light organ that houses the bacteria [58] and might be described by "GO: 0052111 modification by symbiont of host structure", defined as "the process by which an organism effects a change in an anatomical part or cellular component of the host organism" [10] (Figure 2). To describe the growth of *V. fischeri *within the *E. scolopes *light organ, "GO: 0044412 growth or development of symbiont within host" could be used (see Figure 2 for this and the following examples). In the case of *A. diazotrophicus *inside sugarcane, it might be appropriate to use a more specific child term such as "GO: 0075067 growth or development of symbiont in host intercellular space". In either case, "GO: 0033665 regulation of growth or development of symbiont within host" would describe the process by which the symbiont regulates its own growth within the cells or tissues of the host organism [10].

### Fewer structures needed: the case of necrotrophic pathogens

Many symbionts of animal and plant hosts employ a necrotrophic strategy in order to make nutrients available for uptake, by killing the host tissue prior to drawing nutrition from it, e.g. "GO: 0001907 killing by symbiont of host cells" [10]. Some necrotrophs utilize well-differentiated structures for penetration of host tissue, for example appressoria used by fungi and oomycetes [59]. However, differentiated structures such as haustoria are not utilized for nutrition. Instead, emphasis is placed on production of enzymes and toxins for host cell killing [60] and transporters for uptake of catabolized host cell products, e.g. "GO: 0022857 transmembrane transporter activity" and child terms (Figure 2). Toxins produced by necrotrophic phytopathogens may act by triggering programmed cell death in host plant cells, e.g. "GO: 0052042 positive regulation by symbiont of host programmed cell death" (Figure 2). Many GO terms exist to annotate gene products involved in the production, transport, or activity of toxins including: "GO: 0009403 toxin biosynthetic process", "GO: 0015643 toxin binding", "GO: 0019534 toxin transporter activity", "GO: 0009636 response to toxin", "GO: 0010046 response to mycotoxin", and "GO: 0009404 toxin metabolic process" [10]. Furthermore, many GO terms are available for annotating gene products involved in symbiont-induced programmed cell death (see [19] in this supplement). Necrotrophic phytopathogens, including bacteria, fungi and oomycetes, also produce enzymes such as cellulases, xylanases, and pectin-degrading endopolygalacturonases that catalyze degradation of the plant cell wall, e.g. "GO: 0052009 disassembly by symbiont of host cell wall" [61]. In an interesting contrast, necrotrophic animal pathogens such as the oomycete fish pathogen *Saprolegnia parasitica *appear to emphasize secretion of protease inhibitors and proteolytic enzymes [62].

## Summary

An extraordinary diversity of organisms engage in symbiotic interactions, ranging from pathogenic to mutualistic. However, many common themes for fulfilling nutritional requirements have emerged among both hosts and their symbionts. A large number of Gene Ontology terms created by the PAMGO Consortium can be used to identify these commonalities. The more that these terms are used and refined by the community, the more that they will enhance our understanding of multi-organism processes, including mechanisms of nutrient exchange.

## List of abbreviations used

AM: arbuscular mycorrhizal fungus; DAG: directed acyclic graph; GO: Gene Ontology; PAMGO: Plant-Associated Microbe Gene Ontology.

## Competing interests

The authors declare that they have no competing interests.

## Authors' contributions

MCC wrote the manuscript based on discussions with BMT and other PAMGO members. BMT edited the manuscript.

## Supplementary Material

Additional file 1 **Concepts related to symbiotic nutrient exchange, and GO terms for describing associated biological processes and structures**. Most terms in the table are from the "GO: 0008150 biological_process" ontology; those from the "GO: 0005575 cellular_component" ontology are marked with © in the accession field. "Concept" refers to a term commonly employed in the literature. Corresponding GO terms were obtained by querying this concept word against the Gene Ontology using the search function in the GO browser, AmiGO [10]. The rows "Term name", "Accession", "Synonyms", and "Definition" represent GO term fields, found in AmiGO. All biological process terms, but not cellular component terms, also appear in Figure 2. Click here for file
