# Supplementary material for: Common themes in nutrient acquisition by plant symbiotic microbes, described by the Gene Ontology
Source: BMC Microbiol. 2009 Feb 19;9(Suppl 1):S6. doi: 10.1186/1471-2180-9-S1-S6 (PMC2654666; doi:10.1186/1471-2180-9-S1-S6)
Supplement: Additional file 1 — Concepts related to symbiotic nutrient exchange, and GO terms for describing associated biological processes and structures. Most terms in the table are from the "GO: 0008150 biological_process" ontology; those from the "GO: 0005575 cellular_component" ontology are marked with © in the accession field. "Concept" refers to a term commonly employed in the literature. Corresponding GO terms were obtained by querying this concept word against the Gene Ontology using the search function in the GO browser, AmiGO [10]. The rows "Term name", "Accession", "Synonyms", and "Definition" represent GO term fields, found in AmiGO. All biological process terms, but not cellular component terms, also appear in Figure 2. [file 1471-2180-9-S1-S6-S1.doc]

**Additional file 1**

**Concepts related to symbiotic nutrient exchange, and GO terms for describing associated biological processes and structures.**

Most terms in the table are from the “GO : 0008150 biological_process” ontology; those from the “GO : 0005575 cellular_component” ontology are marked with © in the accession field. “Concept” refers to a term commonly employed in the literature. Corresponding GO terms were obtained by querying this concept word against the Gene Ontology using the search function in the GO browser, AmiGO [10]. The rows “Term name”, “Accession”, “Synonyms”, and “Definition” represent GO term fields, found in AmiGO. All biological process terms, but not cellular component terms, also appear in Figure 2.

| Concept | **Arbuscule** |
| --- | --- |
| Term name | Formation by symbiont of arbuscule for nutrient acquisition from host |
| Accession | GO:0075328 |
| Synonyms | None |
| Definition | The assembly by an organism of an arbuscule, a fine, tree-like hyphal structure projected into the host cell for the purpose of obtaining nutrients from its host organism. The host is defined as the larger of the organisms involved in a symbiotic interaction. |
| Notes | Sibling term of “GO : 0052096 formation by symbiont of syncytium involving giant cell for nutrient acquisition from host” and “GO : 0052094 nodule”; child of “GO : 0052093 formation of specialized structure for nutrient acquisition from host”. |
|  |  |
| Concept | **Bacteroid** |
| Term name | Bacteroid-containing symbiosome |
| Accession | GO:0043660 © |
| Synonyms | None |
| Definition | A symbiosome containing any of various structurally modified bacteria, such as those occurring on the root nodules of leguminous plants. |
| Notes | This terms is a child of “GO : 0043659 symbiosome”. |
|  |  |
| Concept | **Bacteroid** |
| Term name | Host bacteroid-containing symbiosome |
| Accession | GO:0043663 © |
| Synonyms | None |
| Definition | A symbiosome containing any of various structurally modified bacteria, such as those occurring on the root nodules of leguminous plants, of a host cell. |
| Notes | Both “GO : 0043660 bacteroid-containing symbiosome” and “GO : 0043663 host bacteroid-containing symbiosome” are child terms that share only the ultimate parent “GO : 0005575 cellular component”. |
|  |  |
| Concept | **Haustorium** |
| Term name | Formation by symbiont of haustorium for nutrient acquisition from host |
| Accession | GO:0052094 |
| Synonyms | Narrow: formation by organism of arbuscule for nutrient acquisition from host  Exact: formation by organism of haustoria for nutrient acquisition from host  Exact: formation by organism of haustorium for nutrient acquisition from host |
| Definition | The assembly by an organism of a haustorium, a projection from a cell or tissue that penetrates the host's tissues for the purpose of obtaining nutrients from its host organism. The host is defined as the larger of the organisms involved in a symbiotic interaction. |
| Notes | A child of “GO : 0052093 formation of specialized structure for nutrient acquisition from host”; not to be confused with appressorium, a fungal structure used in the penetration of host tissues that is characterized by the term “GO : 0075035 maturation of appressorium on or near host”; related terms include “GO : 0075196 adhesion of symbiont haustorium mother cell to host”, “GO : 0075197 formation of symbiont haustorium neck for entry into host”, and “GO : 0075192 haustorium mother cell formation on or near host”. |
|  |  |
| Concept | **Hyphae / mycelium** |
| Term name | Mycelium development |
| Accession | GO:0043581 |
| Synonyms | None |
| Definition | The process whose specific outcome is the progression of the mycelium over time, from its formation to the mature structure. A mycelium is the mass of hyphae that constitutes the vegetative part of a fungus. |
| Notes | A child under “GO : 0032502 developmental process” in the “GO : 0008150 biological_process” ontology. |
|  |  |
| Concept | **Modification of organism during symbiotic interaction** |
| Term name | Modification of morphology or physiology of other organism during symbiotic interaction |
| Accession | GO:0051817 |
| Synonyms | Narrow: regulation of morphology of other organism  Narrow: regulation of physiological process in other organism  Narrow: regulation of physiology of other organism  Exact: modulation of morphology or physiology of other organism during symbiotic interaction  Exact: regulation of morphology or physiology of other organism during symbiotic interaction |
| Definition | The process by which an organism effects a change in the structure or processes of a second organism, where the two organisms are in a symbiotic interaction. |
| Notes | A child term of “GO : 0044419 interspecies interaction between organisms” |
|  |  |
| Concept | **Nodule / Nodulation** |
| Term name | Induction by symbiont in host of tumor, nodule, or growth |
| Accession | GO:0044005 |
| Synonyms | None |
| Definition | The process by which an organism causes the formation of an abnormal mass of cells in its host organism. The host is defined as the larger of the organisms involved in a symbiotic interaction. |
| Notes | A child of “GO : 0044003 modification by symbiont of host morphology or physiology”. |
|  |  |
| Concept | **Nodule / Nodulation** |
| Term name | Nodulation |
| Accession | GO:0009877 |
| Synonyms | None |
| Definition | The formation of nitrogen-fixing root nodules on plant roots. |
| Notes | Allows for symbiont-to-host ammonium transport, e.g. “GO : 0015696 ammonium transport”. |
|  |  |
| Concept | **Nutrient** |
| Term name | Acquisition of nutrients from other organism during symbiotic interaction |
| Accession | GO:0051816 |
| Synonyms | None |
| Definition | The production of structures and/or molecules in an organism that are required for the acquisition and/or utilization of nutrients obtained from a second organism, where the two organisms are in a symbiotic interaction. |
| Notes | A child term of “GO : 0044419 interspecies interaction between organisms” |
|  |  |
| Concept | **Siderophore** |
| Term name | Acquisition by symbiont of nutrients from host via siderophores |
| Accession | GO:0052099 |
| Synonyms | Exact: acquisition by organism of nutrients from host via siderophores |
| Definition | None given |
| Notes | This particular term is a child under “GO : 0044419 interspecies interaction between organisms”, but many more terms exist elsewhere to describe siderophore biosynthesis, transport, and catabolism. |
|  |  |
| Concept | **Symbiosome** |
| Term name | Symbiosome |
| Accession | GO:0043659 © |
| Synonyms | None |
| Definition | A double-enveloped cell compartment, composed of an endosymbiont with its plasmalemma (as inner envelope) and a non-endosymbiotic outer envelope (the perisymbiontic membrane). |
| Notes | This terms is a parent of “GO : 0043660 bacteroid-containing symbiosome”. |
|  |  |
| Concept | **Syncytium** |
| Term name | Formation by symbiont of syncytium involving giant cell for nutrient acquisition from host |
| Accession | GO:0052096 |
| Synonyms | Exact: formation by organism of syncitium involving giant cell for nutrient acquisition from host  Exact: formation by organism of syncytium involving giant cell for nutrient acquisition from host |
| Definition | The assembly by an organism of a syncytium, a nematode-induced multi-nucleate and physiologically active aggregation of fused root cells which exclusively provides the nematode with nourishment during its sedentary life, for the purpose of obtaining nutrients from its host organism. The host is defined as the larger of the organisms involved in a symbiotic interaction. |
| Notes | A child of “GO : 0052093 formation of specialized structure for nutrient acquisition from host”; syncytium in the case of nematodes is distinct from the GO term “GO : 0006949 syncytium formation”, and related terms. The synonyms accommodate alternative spellings of the word “syncytium”. |
